# Effect of Temperature Distribution in Ultrasonically Welded Joints of Copper Wire and Sheet Used for Electrical Contacts

**DOI:** 10.3390/ma11061010

**Published:** 2018-06-14

**Authors:** Jeyaraj Pradeep Kumar

**Affiliations:** Department of Production Engineering, PSG College of Technology, Coimbatore 641 004, Tamil Nadu, India; jp.psgtech@gmail.com; Tel.: +91-944-387-2965

**Keywords:** ultrasonic metal welding, finite element analysis, temperature distribution, strength of the joint in tension, heat flux, friction, plastic deformation, thermocouple

## Abstract

The temperature distribution occurring at the interface while joining a simple electrical contact comprising of a copper wire and a copper sheet using ultrasonic metal welding was analyzed using finite element method. Heat flux due to plastic deformation and friction was calculated and provided as input load for simulation of temperature distribution. The results of temperature obtained from simulation are found to be in good agreement with the results of temperature from experiments measured using thermocouple. Special focus was given to how the heat generated at the wire–sheet interface affect the strength of the joint in tension. With the knowledge of heat generated at the interface while welding, it is possible to control the strength of the joint and produce defect free joints. Based on the results from finite element analysis and experiments, it is observed that the influence of heat developed due to friction and plastic deformation of metallic specimens has a significant effect on the progress of welding and strength of the joint.

## 1. Introduction

Recent technological developments in producing electrical contacts are evolving at a faster rate, resulting in consistent functioning of wide variety of customer durable products. Due to advent of component miniaturization and improvement in manufacturing light weight portable products, there exists a significant demand for superior quality electrical contacts. The electrical and electronic components used in these products are subjected to severe operating conditions during the service life of the products. Ultrasonic metal welding (USMW) has received significant attention in the past few years, and has become a more reliable and suitable process for producing quality electrical contacts to be used in these products. USMW is an environmentally friendly and rapid solid state joining process in which similar or dissimilar metals are joined by the application of ultrasonic vibrations and adequate pressure at the interface. The high frequency relative motion between the parts to be welded result in progressive shearing and plastic deformation between surface asperities. This rubbing action disperses oxides, contaminants and form a sound metallurgical bond between the two metallic parts.

Ding et al. [1] performed thermo-structural numerical analysis on ultrasonic wire bonding to study the effect of bonding parameters on temperature developed at the interface. The authors presented the temperature distribution in wire and the bond pad and reported that the bond force had more effect on development of flash temperature at the contact zone between the interacting asperities and marginal effect on bulk temperature rise developed along the frictionally heated surface. The flash temperature at the asperities between wire and pad on the microscopic scale was found to play a significant role in the formation of solid state bonds. During welding, the temperature at the contact interface increased rapidly at the initial stages of bond formation followed by gradual increase until the end of the vibrations.

Sooriyamoorthy et al. [2] presented a study on temperature distribution at the interface and stress distribution in sonotrode and the welded joint during ultrasonic welding of aluminum sheets. A finite element model was developed for prediction of temperature developed during the process by considering various parameters such as material thickness, clamping force, weld time and coefficient of friction. It was observed that the temperature at the interface increases with increase in weld time and decreases with increase in clamping force. The temperature developed at the interface was measured using thermocouple and the results from experiments were found to be in good agreement with the results from finite element analysis.

Zhao et al. [3] developed thin film thermocouple for measurement of temperature in ultrasonic welding of battery tabs. The dynamic temperature rise during welding was measured using thermocouple with high repeatability. The experimental trials for measurement of temperature were carried out based of design of experiments. The temperature measured by the thermocouple was compared with the temperature measured by an infrared thermal imager and were found to be in good agreement. 

Shin et al. [4] conducted an experimental parametric study on welding of aluminum alloy sheets using ultrasonic spot welding. The process parameters such as vibrational amplitude and weld time were considered in this work. During welding, maximum temperature was observed around the tip of the sonotrode using infrared thermal imager. Significant heat generation occurred at the interface due to frictional vibration and large plastic deformation. The maximum temperature developed for varying vibrational amplitude and weld time was approximately 500 °C and this temperature was about 80% of the melting temperature of the work material. The phenomenon of part marking and part sticking was noticed while welding at higher temperatures.

Elangovan [5] investigated theoretically and experimentally on temperature distribution in ultrasonic welding of copper specimens. Crank Nicolson method was adopted to characterize the unsteady heat transfer scenario at the interface and to determine the temperature developed at each node for varying time steps. It was reported that the temperature at the interface increases with weld time and decreases with increase in thickness of the sheet. It was also inferred that the maximum temperature was obtained in spot welding when compared with seam welding. 

Lee and Cai [6] performed 2-D finite element simulations to understand the effect of four different knurl designs provided in the tip of the sonotrode on strength of the joint. The results from simulation revealed that the sharp knurl design tip severely deform the metallic specimens resulting in reduction of strength of the joint. 

Jedrasiak et al. [7] described a finite element model for prediction of temperature during ultrasonic welding of aluminum–aluminum, aluminum–magnesium and aluminum–steel work materials. The temperature data obtained were used for estimation of growth of intermetallic phase between aluminum-magnesium welds. The K-type thermocouple was embedded in the tip of the sonotrode and the aluminum sheets. The predictive capability of the finite element model was evaluated by comparing results from analysis with the data obtained using thermocouple. 

Chen and Zhang [8] constructed a 3-D finite element model for analyzing the temperature distribution while welding dissimilar automotive alloys. The heat generated due to interface friction was in linear relationship with weld time. The heat generated due to material plastic deformation was found to increase rapidly during the initial phase of the welding and gradually decreases due to decrease in plastic deformation over time. Temperature contours and deformation in shape were obtained using results from simulation. The maximum temperature was located at the interface and the temperature decreases towards the other interfaces such as sonotrode/upper specimen and anvil/lower specimen. High temperature was observed in the upper specimen when compared with the lower specimen. 

De Vries [9] presented the mechanics and mechanism of ultrasonic metal welding of aluminum. The temperature developed at the interface for varying welding conditions were measured using an infrared thermal imager. The temperature at the interface was found to vary 40–80% of the melting temperature of the work material. An empirical relation was developed for calculation of heat flux due to deformation of work material and friction between the parts to be welded.

A comprehensive understanding on formation of joint in ultrasonic metal welding due to heat developed at the interface is still in dispute [10,11,12]. Based on literature survey, research pertaining to correlation of temperature generated at the interface during ultrasonic welding of electrical contacts with strength of the welded joints seems to be not reported. This work was carried out to fill this gap. The primary purposes of this work were to analyze the amount of heat developed during ultrasonic metal welding of an electrical contact comprising of copper wire (100 mm length and 1.2 mm diameter) and copper sheet (100 mm length × 25 mm width × 0.2 mm thickness) and to evaluate the correlation between the heat generated at the interface and strength of the electrical contact joint in tension using finite element analysis and experiments. Finite element analysis was performed using commercially available ABAQUS 6.12 software.

## 2. Experimental Details

The experiments were carried out based on Taguchi’s L9 orthogonal array [13,14] using a conventional lateral drive ultrasonic metal welding machine (National Indosonic, Bangalore, India) (2.5 kW, 20 kHz). Before welding, the weld samples were cleaned with acetone to remove any surface impurity as it may affect the strength of the joint. Based on literature survey, the controllable factors considered in this work for carrying out simulation and experimental trials were the clamping force, amplitude of vibration of the sonotrode and weld time (Table 1). Each factor was varied at three levels. The quality characteristic response variables were the temperature at the interface and the strength of the joint in tension.

**Figure 1:**
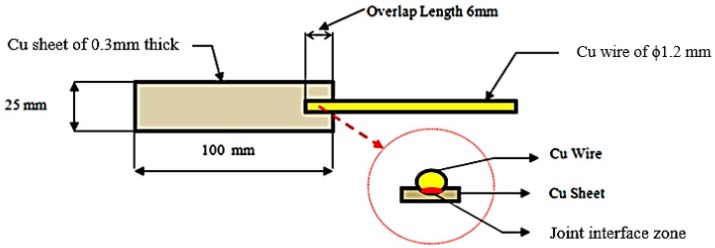
Schematic representation of the joint.

**Figure 2:**
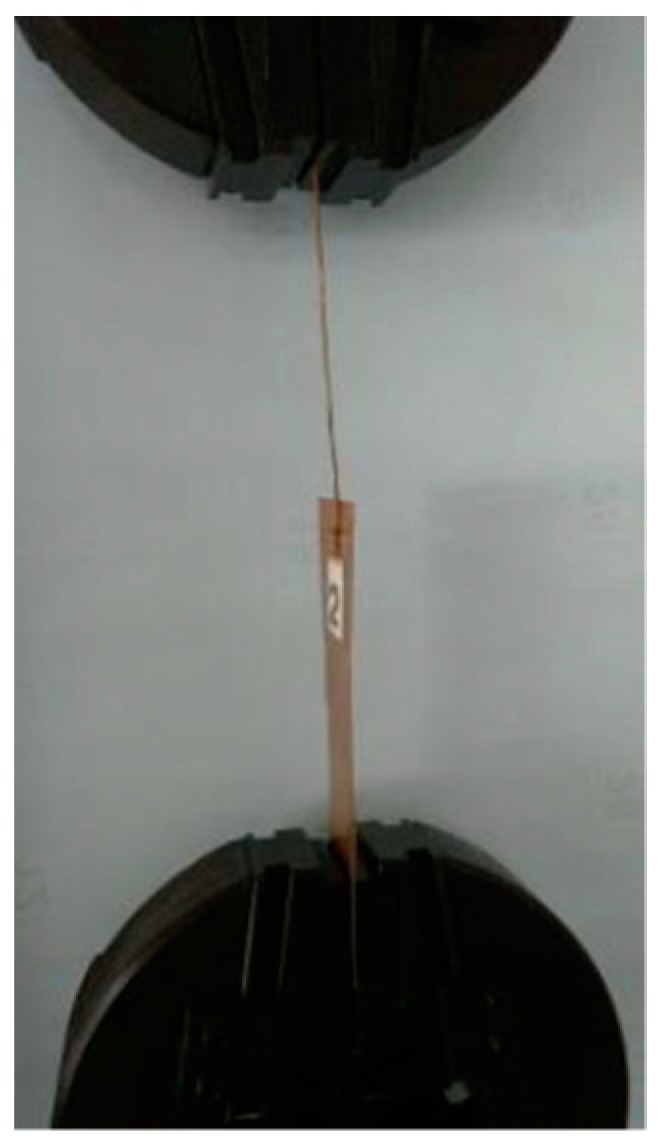
Tensile loading.

A 10 kN tensile testing machine (Hitech, Coimbatore, India) was used to measure the strength of the joint in tension. The schematic representation of the joint and tensile loading condition is shown in Figure 1 and Figure 2.

**Figure 3:**
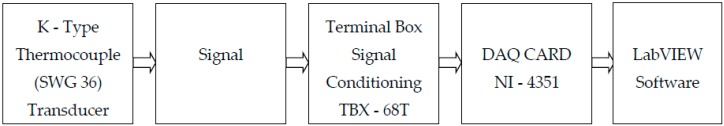
Flow diagram for data transfer in a DAQ system: Temperature.

A suitable instrumentation consisting of K-Type thermocouple (Chromel/Alumel) and data acquisition system (DAQ) interfaced with LabVIEW software (2017, National Instruments, Pune, India) was used to obtain real-time temperature data developed at the interface. Figure 3 depicts the flow diagram of data transfer in data acquisition system.

**Figure 4:**
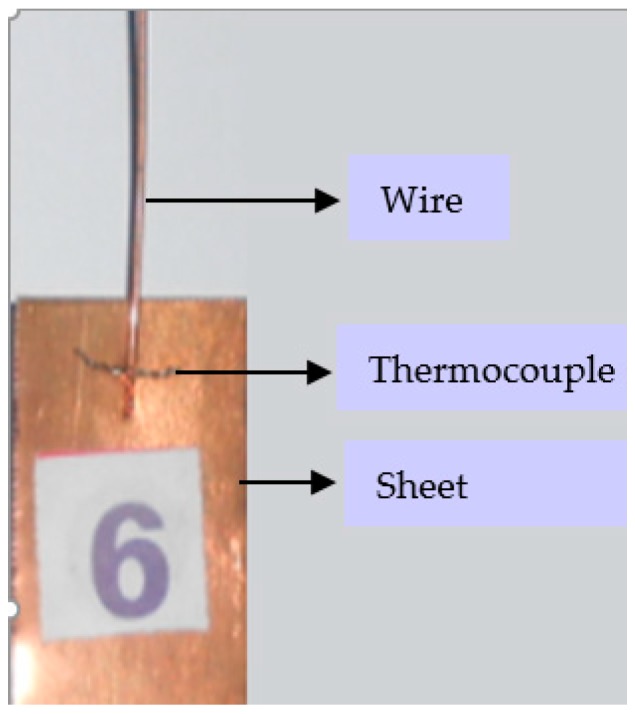
Thermocouple at the interface.

**Figure 5:**
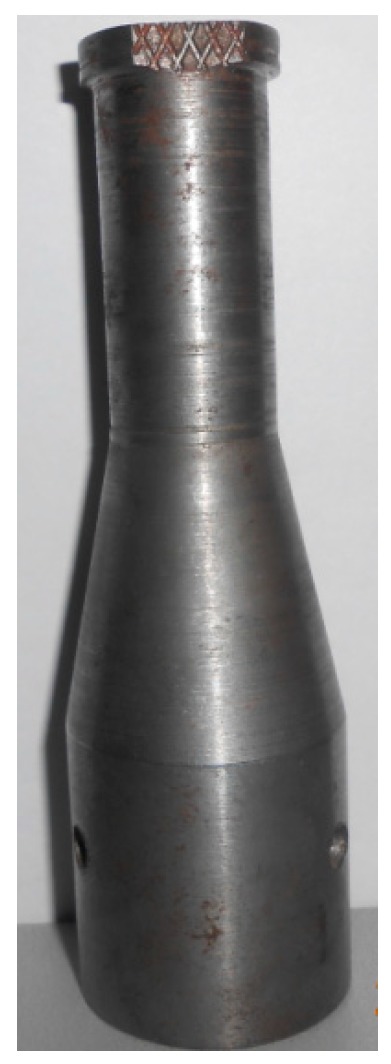
Sonotrode.

The junction of K-Type thermocouple was made with chromel (positive leg: 90% nickel, 10% chromium) and alumel (negative leg: 95% nickel, 2% aluminum, 2% manganese and 1% silicon) wires of SWG 36 (0.193 mm diameter) twisted together for a length of 10 mm. One end of the thermocouple was placed transverse to the longitudinal axis at the interface, as shown in Figure 4, and the other end was connected to the terminal block of the data acquisition system. A 10 Hz notch filter setting was used to optimize measurement frequency and minimize noise level. This DAQ system was interfaced with LabVIEW software to obtain the real-time temperature data developed at the interface. The electrical signal generated from thermocouple was converted to temperature values and displayed in graphical form by LabVIEW software. A custom designed stepped sonotrode with similar circular cross section and gradual change in cross section using a tapered profile at the middle of the sonotrode, as shown in Figure 5, was used to carry out the experiments. The stepped sonotrode has a rectangular tip of 20 mm × 5 mm with serration depth of 0.2 mm, wherein the ultrasonic vibrations and clamping pressure are transmitted to the interface effectively, thereby improving the strength of the joint.

## 3. Finite Element Analysis

### 3.1. CAD Model

**Figure 6:**
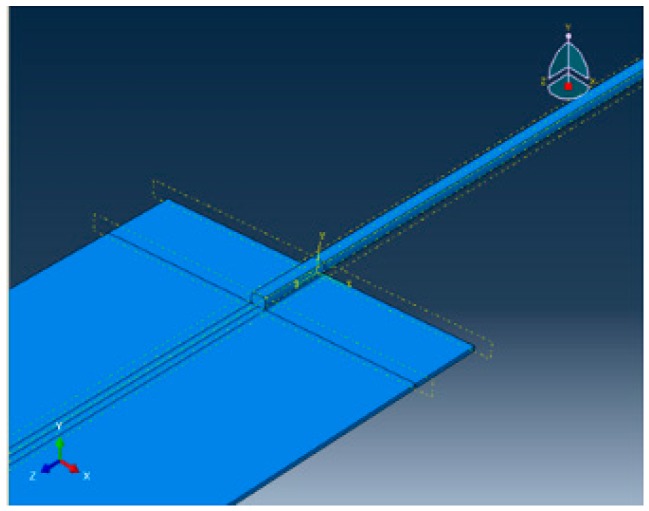
CAD model of the joint.

A CAD model of the joint made of copper wire and copper sheet with an overlap of 6 mm was developed using ABAQUS 6.12 software (Dassault Systems, Vélizy-Villacoublay, France) for performing thermal analysis, as shown in Figure 6.

### 3.2. Material Properties

The material properties considered for thermal analysis are tabulated in Table 2. The properties of copper specimens are taken from ASM Hand Book [15].

### 3.3. Element Selection and Meshing of CAD Model

**Figure 7:**
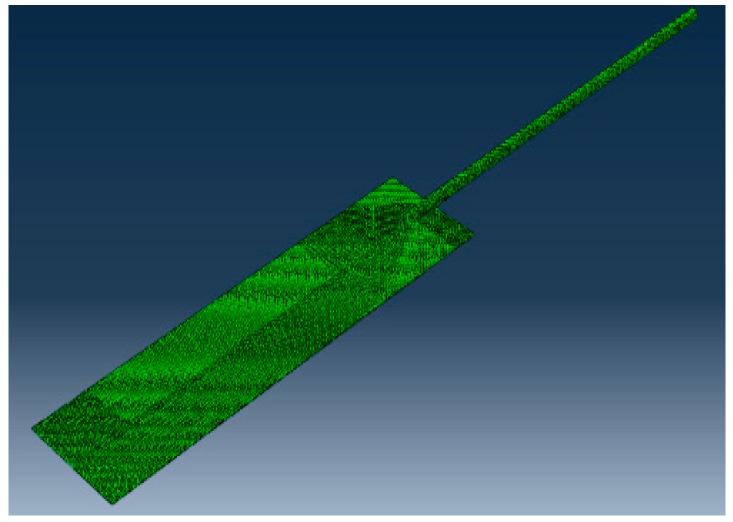
Finite element model of joint.

The specimens were modeled and the element type was selected based on the type of analysis using ABAQUS 6.12 element reference guide [16]. ABAQUS C3D8RT was used for meshing the model. This kind of element is a thermomechanical coupled brick element and has eight-node trilinear displacement and one degree of freedom for temperature with reduced integration scheme and hourglass control. The meshing technique adopted in this study is free meshing which allows the finite element analysis software to generate high quality mesh. A total of 10,400 elements were used for meshing the sheet and 5678 elements were used for meshing the wire based on convergence test and considering the computational intensity and time. The finite element model of the specimen is shown in Figure 7.

### 3.4. Assumptions for Thermal Analysis

The following assumptions were made in simulation of temperature distribution

- Unsteady state is considered for thermal analysis.
- Full contact is established with no air gap between the specimens.
- Room temperature is 30 °C.
- The area in which the friction is effective is assumed to be the area of deformation
- Surfaces exposed to air are set under free convection.

Surface–surface contact was established between wire and sheet. Free convection was applied with overall heat transfer coefficient of 5 W/m^2^·°C [17]. The thermal contact conductance was assumed as 393 W/m^2^·°C [18,19]. The transient analysis was selected with a time step of 0.5 s.

### 3.5. Measurement of Area of Deformation

**Figure 8:**
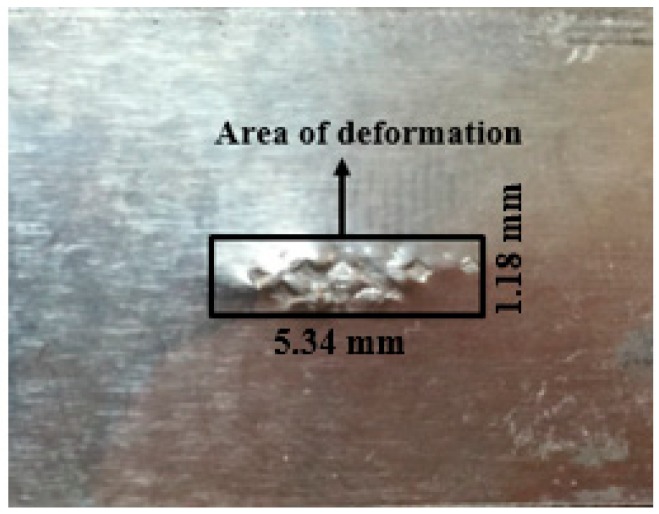
Area of deformation.

In USMW, the upper specimen and the lower specimen are pressed and rubbed against each other to create a solid-state joint. The part of specimens under the tip of sonotrode deform due to static clamping pressure and swaying of specimen surfaces against each other due to ultrasonic vibrations result in formation of joint between the weld specimens. The area of deformation (A_DZ_) play a significant role in calculation of heat flux required for simulation and it is estimated as the rectangular area bordering the interface as shown in Figure 8. The area of deformation for each joint is measured for all the trials of experiments using Tool maker’s microscope (Mitutoya South Asia, NewDelhi, India) [20].

### 3.6. Calculation of Heat Flux

The heat generation due to deformation at the interface is the power dissipated over the weld area. Power at the weld area depends on weld force which is a function of temperature dependent yield strength and the clamping force, as shown in Equation (1).

(1) $$Q_{w}=\frac{P}{A_{w}}=\frac{F_{w}\times V_{\mathrm{avg}}}{A_{w}}$$

where $Q_{w}$ is the heat flux due to deformation in W/m^2^, P is the power in W, $A_{w}$ is the weld area in m^2^, $F_{w}$. is the weld force in N, $V_{\mathrm{avg}}$ is the average sonotrode velocity and equals $4\times \varepsilon_{0}\times f_{w}$, $\varepsilon_{0}$ is the amplitude of vibration of the sonotrode, and $f_{w}$ is the welding frequency. The weld force (F_w_) is given by Equation (2).

(2) $$F_{w}=\sqrt{{\left(\frac{Y_{T}}{2}\right)}^{2}-{\left(\frac{F_{N}/A_{\mathrm{DZ}}}{2}\right)}^{2}}\times A_{\mathrm{DZ}}$$

where Y_T_ is the average temperature dependent yield strength in N/m^2^, F_N_ is the clamping force in N, and A_DZ_ is the area of deformation in m^2^. By substituting Equation (2) into Equation (1), the heat flux due to deformation is obtained as shown in Equation (3).

(3) $$Q_{w}=\frac{\sqrt{{\left(\frac{Y_{T}}{2}\right)}^{2}-{\left(\frac{F_{N}/A_{\mathrm{DZ}}}{2}\right)}^{2}}\times A_{\mathrm{DZ}}\times 4\times \varepsilon_{0}\times f_{w}}{A_{w}}$$

At the end of the welding cycle, the area in which the friction is effective is assumed to be the area of deformation. Thus, A_DZ_ is the A_W_. Applying this condition, the heat flux due to deformation can be calculated, as shown in Equation (4).

(4) $$Q_{w}=\sqrt{{\left(\frac{Y_{T}}{2}\right)}^{2}-{\left(\frac{F_{N}/A_{\mathrm{DZ}}}{2}\right)}^{2}}\times 4\times \varepsilon_{0}\times f_{w}$$

**Figure 9:**
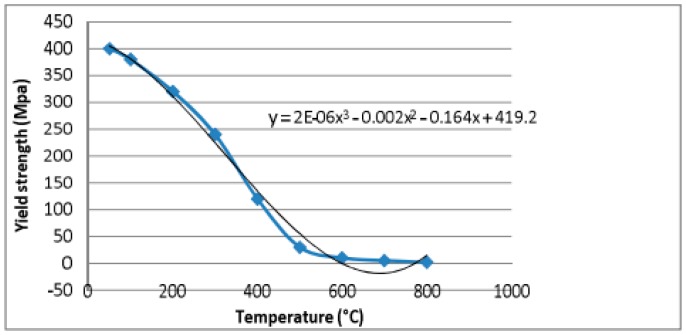
Temperature dependent yield strength of copper.

The temperature dependent yield strength of copper is determined by using third order polynomial equation using the data points available for copper material [15]. The fitted polynomial curve along with equation is shown in Figure 9. The temperature dependent yield strength of copper is calculated using Equation (5).

(5) $$Y_{T}=\frac{\left[\int_{0}^{800}\left({2\times 10}^{-6}T^{3}-0.002T^{2}-0.164T+419.2\right)\times 10^{6}\mathrm{dT}\right]}{\Delta T}$$

where ΔT is the 800 °C. Therefore, $Y_{T}=\frac{\left[\int_{0}^{800}\left({2\times 10}^{-6}T^{3}-0.002T^{2}-0.164T+419.2\right)\times 10^{6}\mathrm{dT}\right]}{800}$ = 182.933 × 10^6^ N/m^2^.

The heat flux due to deformation while joining a copper wire of diameter (1.2 mm) and a copper sheet of thickness (0.2 mm) with clamping pressure of 795 N, amplitude of vibration of the sonotrode of 30 µm, weld time of 2 s and area of deformation of 6.3 × 10^−6^ m^2^, thus obtained as discussed in Section 3.5, is given by

$$Q_{w}=\sqrt{{\left(\frac{182.933\times 10^{6}}{2}\right)}^{2}-{\left(\frac{795/6.3\times 10^{-6}}{2}\right)}^{2}}\times 4\times 30\times 10^{-6}\times 20000=158.92\times 10^{6}\;W/m^{2}$$

Heat flux due to friction ($Q_{\mathrm{FR}}$) is calculated using Equation (6).

(6) $$Q_{\mathrm{FR}}=\frac{\mu \times F_{N}\times 4\times \varepsilon_{0}\times f_{w}}{A_{\mathrm{DZ}}}$$

where µ is the coefficient of friction. Therefore, $Q_{\mathrm{FR}}$ $=\frac{0.3\times 795\times 4\times 30\times 10^{-6}\times 20000}{6.3\times 10^{-6}}$ = 90.85 × 10^6^ W/m^2^

Total heat flux is calculated by adding both heat flux due to deformation and heat flux due to friction. Total heat Flux = Q_W_ + Q_FR_ = 158.92 × 10^6^ W/m^2^ + 90.85 × 10^6^ W/m^2^ = 249.77 × 10^6^ W/m^2^.

## 4. Simulation and Experimental Trials

The simulation and experimental trials were carried out based on parameters provided in Taguchi’s L9 orthogonal array. The heat flux as a function of participating process parameters was calculated and given as input for each simulation trial. The results from simulation and experiments are shown in Table 3.

## 5. Results and Discussions

**Figure 10:**
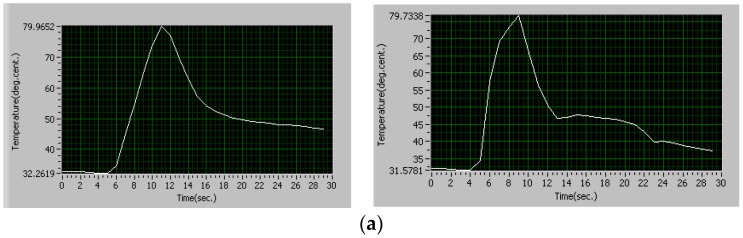

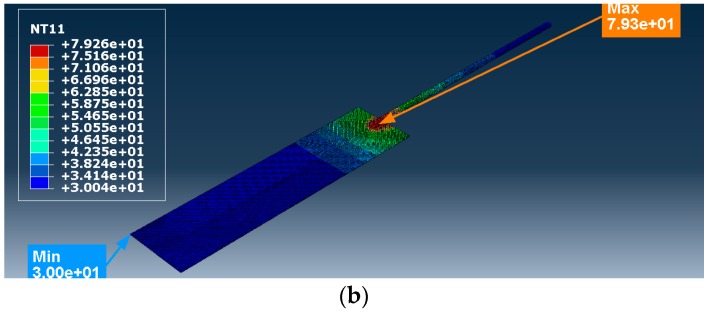
Trial 1, comparison of temperatures (°C): (**a**) results from experiments (Trial 1); and (**b**) result from simulation (Trial 1): Minimum temperature 30.04 °C, Maximum temperature 79.26 °C.

Simulation Trial 1 was carried out with clamping force 795 N, amplitude of vibration of sonotrode 30 µm and weld time 2 s. The process parameters in this trial were set at lower level. The heat flux calculated for this combination of process parameters is 249.77 × 10^6^ W/m^2^. The temperature obtained from simulation is 79.26 °C. The average temperature obtained from experiments for the same combination of process parameter using thermocouple is 79.85 °C. The strength of the joint in tension obtained is 187.721 N. As all the process parameter values are set at the lower levels, the temperature developed at the interface and the strength of the joint obtained in this trial is minimum when compared with all the other trials. The results from simulation and experiments are shown in Figure 10.

**Figure 11:**
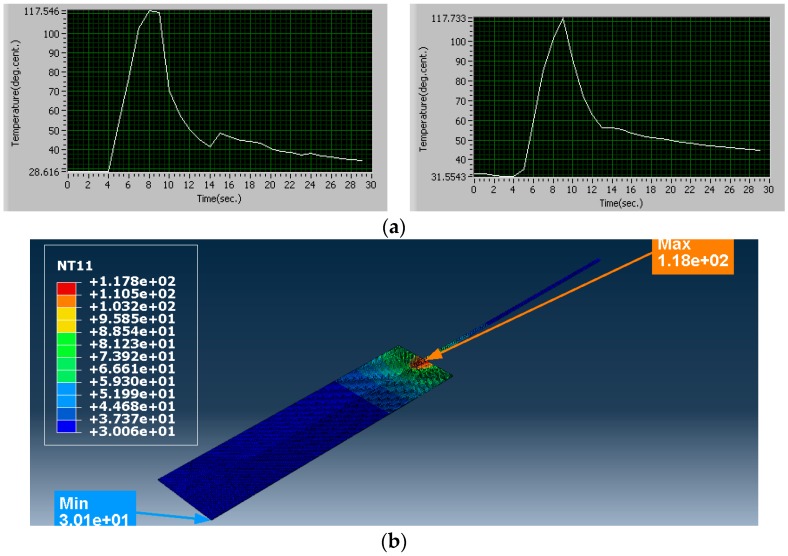
Trial 5, comparison of temperatures (°C): (**a**) results from experiments (Trial 5); and (**b**) result from simulation (Trial 5): Minimum temperature 30.06 °C, Maximum temperature 117.8 °C.

Simulation Trial 5 was carried out with clamping force 995 N, amplitude of vibration of sonotrode 42.5 µm and weld time 2.5 s. The process parameters in this trial were set at medium level. The heat flux calculated for this combination of process parameters is 355.73 × 10^6^ W/m^2^. The temperature obtained from simulation is 117.8 °C. The average temperature obtained from experiments for the same combination of process parameter is 117.64 °C. The strength of the joint obtained is 213.342 N. The results from simulation and experiments are shown in Figure 11.

**Figure 12:**
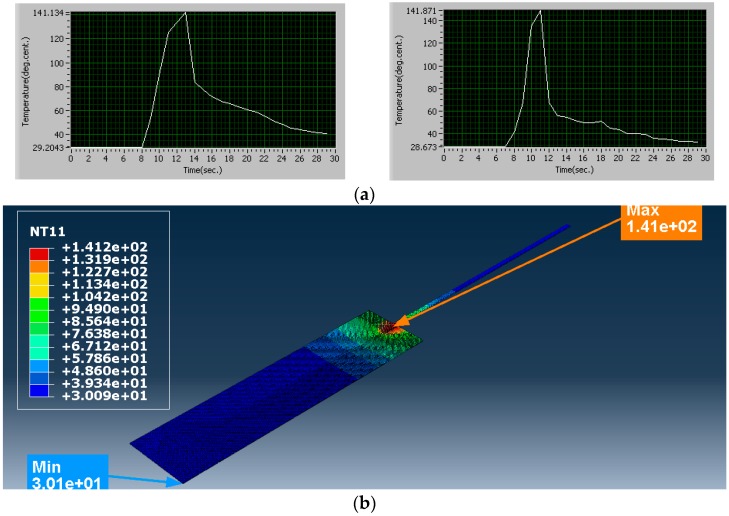
Trial 9, comparison of temperatures (°C): (**a**) results from experiments (Trial 9); and (**b**) result from simulation (Trial 9): Minimum temperature 30.09 °C, Maximum temperature 141.12 °C.

Simulation Trial 9 was carried out with clamping force 1195 N, amplitude of vibration of sonotrode 57 µm and weld time 3 s. The process parameters in this trial were set at higher level. The heat flux calculated for this combination of process parameters is 483.5 × 10^6^ W/m^2^. The temperature obtained from simulation is 141.2 °C. The average temperature obtained from experiments for the same combination of process parameter is 141.50 °C. The strength of the joint obtained is 231.432 N. As all the process parameter values were at higher levels, the temperature developed at the interface and the strength of the joint obtained in this trial is maximum when compared with all the other trials. The results from simulation and experiments are shown in Figure 12.

In Simulation Trials 1, 4 and 7, the clamping force and the weld time were set constant at 795 N and 2 s, respectively, whereas the amplitude of vibration of the sonotrode was varied at 30 µm, 42.5 µm and 57 µm. The temperature generated at the interface in these trials are 79.26 °C, 83.27 °C and 99.16 °C, respectively, as shown in Table 3. Based on the results, it is observed that the temperature at the interface increases with increase in amplitude. This is in agreement with the observations from the experiments. Increase in amplitude of vibration of the sonotrode results in increased sliding action between the specimens leading to plastic deformation and rise in temperature. Similar trend was observed with varying amplitude of vibration of the sonotrode in Simulation Trials 2, 5 and 8 where the clamping force and weld time were set constant at medium level (995 N, 2.5 s) and in Simulation Trials 3, 6 and 9 where the clamping force and weld time were set constant at higher level (1195 N, 3 s). 

Simulation Trials 1,5 and 9 correspond to lower level (795 N, 30 µm and 2 s), medium level (995 N, 42.5 µm, 2.5 s) and higher level (1195 N, 57 µm and 3 s) of process parameters, respectively. The temperature generated at the interface in these trials are 79.26 °C, 117.8 °C and 141.2 °C, respectively, as shown in Figure 10, Figure 11 and Figure 12. The strength of the joints obtained in these trials are 187.721 N, 213.342 N and 231.432 N, respectively, as shown in Table 3. Based on the results, it is observed that higher level parameters produce maximum strength of the joint in tension of 231.432 N when compared with lower level and medium level combinations of process parameters.

**Figure 13:**
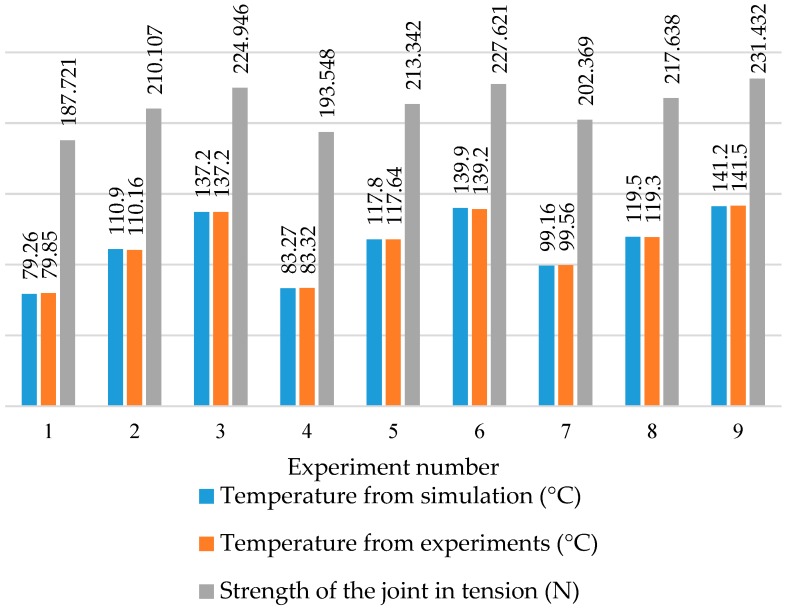
Comparison of temperature and strength of the joint under tensile loading.

The results from simulation were compared with results from experiments, as shown in Figure 13. The temperature obtained from simulation is found to be in good agreement with the temperature from experiments measured using thermocouple. Therefore, the developed finite element analysis model is found useful to predict the temperature. It is also observed in Figure 13 that the strength of the joint under tensile loading correlate well with the temperature. The co-efficient of correlation between temperature and strength of the joint in tension is determined as 0.99. Hence, it is observed the temperature at the interface has significant effect on progress of the welding and strength of the joint.

## 6. Conclusions

Temperature distribution while joining metallic wire and a sheet made of copper was analyzed using finite element analysis. The results of the analysis reveal the following salient observations.

- The results from simulation and experiments conducted based on Taguchi’s L9 orthogonal array reveal that the maximum temperature developed during welding is less than the melting point of the work material, validating that the USMW is a solid state welding process.
- It is observed from the analysis that the influence of heat generated due to deformation and friction is significant in the process of formation of joint. The results of temperature from simulation are found to be in good agreement with results of temperature from experiments measured using thermocouple. Thus, the developed finite element model is validated.
- The results of temperature developed at the interface are compared with results of strength of the joint under tensile loading. It is inferred that the strength of the joint correlate well with the temperature developed at the interface indicating that the temperature at the interface has significant effect on strength of the joint. It is observed that the strength of the joint depends on the variations of heat generated during welding under different process parametric conditions.

## Figures and Tables

**Table 1:** Factors and levels.

Factors	Units	Designation	Level 1	Level 2	Level 3
Clamping force	N	A	795	995	1195
Amplitude of vibration of sonotrode	µm	B	30	42.5	57
Weld time	second	C	2	2.5	3

**Table 2:** Material properties of copper.

Properties	Value
Young’s Modulus (GPa)	115
Poisson ratio	0.3
Density (kg/m^3^)	8940
Thermal conductivity (W/m °C)	391
Specific heat (J/Kg °C)	385
Thermal expansion co-efficient(°C^−1^)	1.66 × 10^−5^

**Table 3:** Results of temperature from simulation and experiments.

Trial No.	Clamping Force (N)	Amplitude of Vibration of Sonotrode (μm)	Weld Time (s)	Temperature from Simulation (°C)	Temperature from Experiments (°C)			Strength of the Joint in Tension * (N)
					Trial 1	Trial 2	Average	
1	795	30	2	79.26	79.97	79.73	79.85	187.721
2	995	30	2.5	110.9	110.06	110.25	110.16	210.107
3	1195	30	3	137.2	137.38	137.01	137.20	224.946
4	795	42.5	2	83.27	83.25	83.39	83.32	193.548
5	995	42.5	2.5	117.8	117.55	117.73	117.64	213.342
6	1195	42.5	3	139.9	139.01	139.38	139.20	227.621
7	795	57	2	99.16	99.74	99.37	99.56	202.369
8	995	57	2.5	119.5	119.60	119.00	119.30	217.638
9	1195	57	3	141.2	141.13	141.87	141.50	231.432

* Average of two experimental trials.
